# A Nordic Perspective on Patient Online Record Access and the European Health Data Space

**DOI:** 10.2196/49084

**Published:** 2024-06-27

**Authors:** Maria Hägglund, Anna Kharko, Annika Bärkås, Charlotte Blease, Åsa Cajander, Catherine DesRoches, Asbjørn Johansen Fagerlund, Josefin Hagström, Isto Huvila, Iiris Hörhammer, Bridget Kane, Gunnar O Klein, Eli Kristiansen, Jonas Moll, Irene Muli, Hanife Rexhepi, Sara Riggare, Peeter Ross, Isabella Scandurra, Saija Simola, Hedvig Soone, Bo Wang, Maedeh Ghorbanian Zolbin, Rose-Mharie Åhlfeldt, Sari Kujala, Monika Alise Johansen

**Affiliations:** 1 Participatory eHealth and Health Data Research Group Department of Women's and Children's Health Uppsala University Uppsala Sweden; 2 Medtech Science & Innovation Centre Uppsala University Hospital Uppsala Sweden; 3 School of Psychology Faculty of Health University of Plymouth Plymouth United Kingdom; 4 Division of General Medicine, Department of Medicine Beth Israel Deaconess Medical Center Harvard Medical School Boston, MA United States; 5 Department of Information Technology Uppsala University Uppsala Sweden; 6 Norwegian Centre for E-Health Research University Hospital of North Norway Tromsø Norway; 7 Department of ALM Uppsala University Uppsala Sweden; 8 Department of Computer Science Aalto University Espoo Finland; 9 Business School Karlstad University Karlstad Sweden; 10 Centre for Empirical Research on Information Systems School of Business Örebro University Örebro Sweden; 11 School of Informatics University of Skövde Skövde Sweden; 12 E-Medicine Centre Department of Health Technologies Tallinn University of Technology Tallinn Estonia; 13 Research Department East Tallinn Central Hospital Tallinn Estonia

**Keywords:** patients’ online record access, open notes, electronic health records, EHR, patient portals, European Health Data Space, digital health, health care, patient access

## Abstract

The Nordic countries are, together with the United States, forerunners in online record access (ORA), which has now become widespread. The importance of accessible and structured health data has also been highlighted by policy makers internationally. To ensure the full realization of ORA’s potential in the short and long term, there is a pressing need to study ORA from a cross-disciplinary, clinical, humanistic, and social sciences perspective that looks beyond strictly technical aspects. In this viewpoint paper, we explore the policy changes in the European Health Data Space (EHDS) proposal to advance ORA across the European Union, informed by our research in a Nordic-led project that carries out the first of its kind, large-scale international investigation of patients’ ORA—NORDeHEALTH (Nordic eHealth for Patients: Benchmarking and Developing for the Future). We argue that the EHDS proposal will pave the way for patients to access and control third-party access to their electronic health records. In our analysis of the proposal, we have identified five key principles for ORA: (1) the right to access, (2) proxy access, (3) patient input of their own data, (4) error and omission rectification, and (5) access control. ORA implementation today is fragmented throughout Europe, and the EHDS proposal aims to ensure all European citizens have equal online access to their health data. However, we argue that in order to implement the EHDS, we need more research evidence on the key ORA principles we have identified in our analysis. Results from the NORDeHEALTH project provide some of that evidence, but we have also identified important knowledge gaps that still need further exploration.

## Introduction

The digitalization of health care is increasing rapidly, changing the way patients communicate and collaborate with health care providers. The importance of access to and use of digital health data became even more evident during the COVID-19 pandemic [1], including the use of online patient portals and patients’ online record access (ORA) [2]. The Nordic countries are, together with the United States, forerunners in providing their residents with online tools that enable interaction not only with health care but also with the patient’s own health data [3]. ORA has become a key means to these ends [4-6]. National patient portals have been implemented in all Nordic countries, enabling residents to access different health-related e-services, for example, patient-accessible electronic health records (PAEHRs). In the United States, legislation mandates patients to have ORA [7]. Textbox 1 provides an overview of key concepts related to patients’ ORA that will be used in this paper.

**Key terminology.**

**Electronic health record (EHR)**

The World Health Organization defines EHRs as *“*shared patient records that contain historical data about a patient that are compiled from all local Electronic Medical Records” [8].

**Patient-accessible EHR (PAEHR)**

PAEHRs are online services providing patients secure access to view and sometimes edit or comment on their electronic health records (EHRs) made available by their health care providers [5], that is, online record access (ORA).

**European Health Data Space (EHDS)**

The EHDS is a health-specific ecosystem comprising rules, common standards and practices, infrastructure, and a governance framework [9].

**Open notes**

Online access to the visit note summaries, or the narrative, free-text entries, written by clinicians about patient health.

**ORA**

ORA has been used as a “solution-neutral” concept to describe the phenomenon of patients’ online record access [10]. ORA can be implemented through a PAEHR or any other technical system that gives patients access to their health records online.

**Patient portal**

Patient portals are online portals that can be provided locally by a specific health care provider or nationally, as is the case in the Nordic countries. Patient portals are increasingly used to provide patients with ORA. In some patient portals, a PAEHR is provided as a specific service [11], whereas others may have more seamlessly integrated ORA through different patient portal functions. In a local patient portal, patients often have ORA to only 1 specific EHR system, whereas national patient portals can provide ORA to several EHR systems.

In many countries, ORA is considered a logical extension of patients' already existing legal rights to request copies of their health records. ORA provides a rapid and convenient method of accessing the information held by clinicians that increases the total number of patients who read their records. Considering the growing body of evidence presenting the benefits of ORA for the individual (in terms of improved health outcomes and self-management) [4-6,12,13], we argue that health organizations in other countries can learn from the Nordic experience and should also consider striving to provide patients’ ORA [14].

In parallel with the increased use of digital health services, the importance of accessible and structured health data has also been highlighted by policy makers internationally. In the United States, a federal rule in the 21st Century Cures Act mandates that US health care providers offer patients access, with few exceptions, to all the health information in their electronic medical records without charge [7]. The 21st Century Cures Act is also motivated by the idea of a health app economy, and it is mandated that patients’ health information be available in a form that is downloadable to third-party apps. In Europe, the proposal for an EHDS aims to both empower people to control and use their health data in their home country or other member states, as well as offer “a consistent, trustworthy and efficient framework to use health data for research, innovation, policy making, and regulatory activities, while ensuring full compliance with the EU's high data protection standards” [9]. Furthermore, the EU has adopted the NIS directive (EU 2022/255), which sets requirements for security in networks and information systems. The rules cover providers of socially important services and certain digital services where health care is a designated sector [15].

Despite growing international evidence that ORA has the potential to empower patients and yield many health benefits, its implementation has not always been straightforward [16,17]. Not all patients use online portals [4,5,12,18], and ORA remains controversial among providers [10,19]. Health care professionals have raised concerns regarding patient ORA in several contexts where it has been implemented [10,20-23]. The concerns include that patients might misunderstand what they read and become worried, and that clinicians’ workload will increase as patients ask more questions, both during and between appointments. Within mental health care, such concerns have been especially prominent [19,22,24].

Despite clinicians’ concerns and the need for a more fine-grained policy concerning, for example, proxy access (when an informal caregiver, such as a family member, has ORA on behalf of a patient) [25], psychiatric care [26], and ethical exemptions from ORA [27], mounting international experience challenges clinicians’ skepticism and evinces the benefits to patients of this practice innovation [28]. Nonetheless, as the shift toward giving patients more autonomy over their health data is underway, there is an urgent need to address more contested aspects of ORA. Doing so may simultaneously offer guidance to other countries, where implementation is currently lagging behind. We argue that to ensure the full realization of its potential in the short and long term, there is a pressing need to study patients’ ORA from a cross-disciplinary, technical, clinical, humanistic, and social science perspective that looks beyond narrow technical aspects of implementation [29]. A project that aims to do this is the NORDeHEALTH research project, launched in 2021 [3]. NORDeHEALTH focuses on studying novel digital services and innovation, exploring different ways to make national patient portals and patients’ ORA more useful to both patients and health care professionals, supporting person-centered care, patient self-management, and empowerment, as well as collaboration.

In this viewpoint paper, we will summarize the key policy changes in the EHDS with relevance to ORA and discuss the proposed changes in the context of the latest research findings from the Nordic Region through the NORDeHEALTH project.

## The European Health Data Space and Patient Online Record Access

The EHDS proposal aims to “improve access to, and control by, natural persons over their personal electronic health data in the context of health care (primary use of electronic health data), as well as for other purposes that would benefit the society such as research, innovation, policy-making, patient safety, personalized medicine, official statistics or regulatory activities (secondary use of health data)” [9]. “Natural person” is a legal term used to signify an individual human being, distinguishing them from a “juridical person,” which can encompass other entities too. We will use “person” in our text to signify a “natural person,” unless in a direct quote from the EHDS proposal. Negotiations among EU member states have been completed, and in April 2024, the European Parliament adopted the EHDS proposal. Once the new EHDS regulation is formally adopted, which is expected to be in autumn 2024, it will become applicable in different stages according to use case and data type, allowing member states time to adapt to the regulation [30].

Primary use of electronic health data is the main focus of patients’ ORA and is further defined as the processing of personal electronic health data for the provision of health services to assess, maintain, or restore the state of health of the natural person to whom that data relates, including the prescription, dispensation, and provision of medicinal products and medical devices, as well as for relevant social security, administrative, or reimbursement services.

Embedded within the EHDS Chapter 2, Primary use of electronic health data, Section 1, Article 3 describes the “rights of natural persons in relation to the primary use of their personal electronic health data” [9]. In our analysis of the EHDS proposal, we have identified 5 key principles of high relevance for patient ORA, where results from the NORDeHEALTH project may contribute to the design and implementation of the EHDS across Europe. The five principles we have identified are (1) the right to access, (2) proxy access, (3) patient input of their own data, (4) error and omission rectification, and (5) access control.

## Overview of the NORDeHEALTH Research Project

The NORDeHEALTH project, funded by NordForsk (grant 100477), has research partners in Sweden, Norway, Finland, Estonia, and the United States. The goal is to enable further digitalization of the public health sector by providing concrete feedback to the national authorities in the respective countries and providing guidelines and frameworks for the design, implementation, and evaluation of patients' ORA as well as other eHealth services.

The foundation for the NORDeHEALTH research project is a sociotechnical analysis of the context in the respective country using a model proposed previously [29,31]. The model defines eight dimensions that are essential to consider when designing and implementing sociotechnical systems in health care: (1) hardware and software, (2) clinical content, (3) human-computer interface, (4) people, (5) workflow and communication, (6) internal organizational policies, procedures, and culture, (7) external rules, regulations, and pressures, and (8) system measurement and monitoring [31]. Key focus areas in the NORDeHEALTH research project have included policy and regulations for patients’ ORA in mental health care [32-34], ORA for adolescents and parents [25,35], and ORA within oncology, specifically focusing on multidisciplinary team conferences. The project also investigates benchmarking for the usability and acceptance of national patient portals and patients’ ORA [6,36], which iteratively feeds into the co-design of novel ORA and patient portal functionality.

Empirical data in the project is gathered by exploring the needs of specific patient and demographic groups using the current implementations of PAEHRs as a case; patients with mental health disorders, patients with cancer, and adolescents and their caregivers. Most research into patient ORA to date collects data from one country or region [4-6,12], making it difficult to compare results across contexts. In the NORDeHEALTH project, we therefore designed an international cross-sectional survey study, and in 2022, data were collected simultaneously in Sweden, Norway, Finland, and Estonia [37]. The survey aimed to study patients’ experiences with the PAEHR provided through the national patient portals in the respective countries.

Textbox 2 provides an overview of how the NORDeHEALTH research contributes to the patient ORA principles we have identified in the EHDS proposal: (1) the right to access, (2) proxy access, (3) patient input of their own data, (4) rectifying errors and omissions, and (5) access control. In the next sections, we further deepen the analysis of the 5 principles.

**Online record access (ORA) principles in the context of recent research from the Nordics.**

**The right to access**

- Despite the many similarities, ORA implementation varies in the Nordic region.
- Patients’ experiences depend on platform usability.

**Proxy access**

- Current regulations for parental and adolescent proxy access greatly differ between the Nordic countries.
- Proxy access other than parental has even greater variation, and is not allowed in, eg, Sweden.

**Patient input of their own data**

- Despite the advanced stage of ORA in the Nordic countries, patient input is not widely available.
- Nordic patients have expressed repeated interest in the ability to contribute to their record.

**Error and omission rectification**

- A high number of Nordic patients find serious errors in their records through ORA.
- Some groups of patients report more errors than others.
- At present, only a minority of Nordic patients attempt to rectify the errors.

**Access control**

- A minority of Nordic patients have reported unwanted access to their electronic health records

## The Right to Access

### The Proposal

The EHDS proposal clearly states that “natural persons shall have the right to access their personal electronic health data” immediately, free of charge, and in an easily readable, consolidated, and accessible form [9]. This is not limited to electronic health records (EHRs) data, but considering the EHR's core role for documentation in health care, patients' ORA must be considered essential for EHDS. Article 3, paragraph 2, continues to declare that:

natural persons shall have the right to receive an electronic copy, in the European electronic health record exchange format […] of at least their electronic health data in the priority categories referred to in Article 5 [9].

These include patient summaries, electronic prescriptions, electronic dispensations, medical images and image reports, laboratory results, and discharge reports.

### The Research

Many European countries already have legislation stipulating that patients should have ORA [16]. In Norway, the patient is both the object and the owner of the health record. The Norwegian Patient Rights Act of 2001 states that patients have the right by law to access their health records [38], and in 2013, a White Paper stated that patients should have digital access [39]. Similar legislation is in place in all the Nordic countries [16].

In Germany, the Patient’s Rights Act of 2013 stipulates that health care professionals must document diagnosis and treatment promptly and comprehensively. It grants patients the right to fully view their records and attain an electronic copy [40], yet progress in implementing patients’ ORA has been slow. In the Netherlands, patients have had the right to a digital copy of all the information in their EHRs since 2020 [17], and different types of incentive programs have been implemented to encourage health care providers to provide such access.

Given the current challenges in implementing patients' full ORA across Europe, the EHDS proposal is ambitious. Mandating patients’ ORA is to be broadly encouraged, considering the positive experiences reported by patients with full ORA, but experiences show that regulations are often not enough to ensure implementation.

The NORDeHEALTH project has designed and tested a sociotechnical framework for studying and comparing factors that affect the implementation and adoption of patient ORA. The framework was designed based on the existing Sittig and Singh sociotechnical framework [31]. ORA-specific factors are explored related to, for example, what information patients have access to and when they can access it (immediately or with a delay), what functionality is provided (eg, being able to upload or edit information, proxy access), rules and regulations for ORA (on national or local levels), the usability of the PAEHR, technical infrastructure, and population characteristics (eg, educational levels, digital literacy, and diversity). An in-depth understanding of the local sociotechnical context is essential for comprehending the impact of ORA and being able to design successful interventions for implementing ORA. A study on the implementation of ORA in Sweden and the Netherlands identified resistance from health care professionals and technical infrastructure challenges as main barriers, whereas existing national infrastructure and program management, strong leadership, and stakeholder engagement (including both patients and health care professionals) were identified as success factors [17].

### Viewpoint

We argue that simply enabling ORA is not enough to ensure that all patients can use it. Usability is a key factor in the adoption of ORA [41]. Therefore, the NORDeHEALTH project strives to benchmark PAEHR usability [6] and investigate how it affects the acceptance and adoption of PAEHRs among different patient groups.

## Proxy Access

### The Proposal

In the EHDS proposal, proxy access is described in Article 3, paragraph 5. Member States shall:

establish one or more proxy services enabling a natural person to authorize other natural persons of their choice to access the electronic health data on their behalf [9].

This includes guardians and other representatives, either automatically or upon request.

### The Research

With a growing population of older people (>60 years) seeking health care services, many of whom are likely to have (multiple) chronic conditions, the demands on health and social care services are increasing [42]. However, the time that individuals with chronic conditions seek health and social care represents only a fragment of their 24/7 lived experience of coping with a chronic disease. As we grow older, we often become increasingly dependent on psychosocial and physical support outside of formal health care services, but this is far from only an issue for older people. Patients with cognitive or physical disabilities often rely on family or informal caregivers for support in managing their health. Parents, especially of children with chronic or life-threatening conditions, have an instrumental role in their children’s care and report great benefits from ORA [35] when it is available. Having a (strong) social network and informal caregivers (eg, family and friends), especially in times of life-changing illness, could mean the difference between survival and death [43]

Despite informal caregiving being an essential part of health care, it is rarely given a lot of credit. In fact, the vast majority of caregiving is informal, and it is undertaken by family members free of charge and with no support provided for them, often at great burden [44,45]. Thus, health care outcomes highly depend on the competence and ability of informal caregivers. Still, informal caregivers are often left out of the conversation [46], not the least when digitalization is introduced, and informal communication needs to be formalized.

In the NORDeHEALTH project, research into proxy access focuses on how parental ORA differs between the participating countries [25] and what this could mean for streamlining proxy access across Europe. Further studies on parental proxy access and adolescents’ ability to deny access in certain situations are in process. For general proxy access, there is even greater diversity across countries and even less research available. Internationally, when patients are given online access to their records, they are often given the option to share their records with a proxy if needed, usually a close family member such as a spouse or adult son or daughter [47]. In a US study, 2 out of 3 surveyed hospitals offered adult patients the option of granting portal access to an informal caregiver, but among hospitals that did, the process for obtaining proxy credentials was often difficult and time-consuming [48]. In the original Swedish implementation of ORA, patients could assign a proxy to be able to access their records. This function was available after secure log-in to the record, and the patient could assign access to any person in Sweden by adding the social security number of the person and choosing what parts of the PAEHR to share [49]. Despite this flexibility of the solution, the Swedish Authority for Privacy Protection requested the function be shut down, and after several appeals from Region Uppsala, the Supreme Administrative Court in Sweden finally decided to prohibit the function where patients can share their information with others, finding it to be in conflict with the Patient Data Act (a part of the Swedish Data Protection Act (2018:218) and the Swedish Data Protection Regulation (2018:219) that entered into force on May 25, 2018 [50]), which refers to allowing only patients themselves direct access to their medical records—not someone else [49].

### Viewpoint

In order to implement the EHDS proposal, it will be essential to streamline regulations for, and implementation of, proxy access across Europe. Acknowledging that different types of proxy access exist and come with their own set of challenges will also be important, distinguishing, for example, parental proxy access from other forms of proxy access.

## Rectifying Errors and Omissions

### The Proposal

The EHDS proposal, Article 3 §7, stresses that Member States shall ensure that:

*natural persons can easily request rectification online* [9]

### The Research

In a US study of 22,000 patients who read their notes, 1 in 5 reported finding an error, and 40% of those perceived the error to be serious [51]. The most common errors were related to diagnoses, medical histories, medications, test results, notes on the wrong patient, and notes pertaining to the wrong side of the patient’s body (left vs right). Erroneous records may contribute to diagnostic errors that are common in health care [52]. Between 1 in 20 and 1 in every 6 medical consultations results in missed, wrong, or delayed diagnoses [53]. Most diagnostic errors relate to common conditions such as congenital heart failure, pneumonia, and urinary tract infections [54]. Research also shows repeated, missed opportunities to detect cancer [55,56].

Patients have so far had a marginal role in diagnostic processes, as acknowledged in the US National Academy of Medicine’s report “Improving Diagnosis in Health Care.” The report prompts a deeper discussion about the role of patients in closing feedback loops in care and helping to avoid mistakes that can lead to diagnostic errors, and ultimately patient harm [57]. Patient ORA is cited as a mechanism for improving diagnostic accuracy [57] and has been described by medical safety experts as a “transforming concept” in patient safety [58]. Emerging research supports these conclusions [13,59,60]. Patient ORA may help patients avoid delays and missed diagnoses by encouraging timely follow-up of recommended tests, results, and referrals [51]. Patients with ORA who identify and report errors could prevent clinicians from relying on incorrect data that may lead to poor diagnostic or treatment decisions or even legal liability [61]. A meta-analysis of 20 ORA-related randomized clinical trials (involving 17,387 patients) supports the conclusion that ORA could improve patient safety [13]. Most research to date on patients’ ORA and documentation errors has been performed in the United States with a remarkably different medico-legal system from the European one. In this respect, the NORDeHEALTH research complements the existing research and provides important evidence for the usefulness of patient ORA in patient safety work in contexts dominated by public health care provision.

In addition, most studies focus on somatic care and exclude mental health care. A recent NORDeHEALTH study made a comparison between patients who had received mental health care (the MHC group) and patients who had not (non-MHC group), regarding their experiences of finding errors or missing information in their online records [62]. MHC respondents (n=3131) experienced errors (MHC 1586/3131, 50.65% and non-MHC 3311/9203, 35.98%) and omissions (MHC 1089/3131, 34.78% and non-MHC 2427/9203, 26.37%) in the EHR at a higher rate compared with non-MHC respondents (n=9203). The statistically significant differences between the MHC and non-MHC groups remained when comparing a stratified subgroup sample adjusted for age and gender [62].

### Viewpoint

In the NORDeHEALTH patient survey [37], we explore the extent to which patients in Sweden, Norway, Finland, and Estonia find errors or missing information in their PAEHR and the action they have taken in these situations [62]. The results from these studies will help guide the further implementation of the EHDS with respect to the management of errors and missing information.

## Patients’ Input of Their Own Data

### The Proposal

Article 3, §6 of the EHDS proposal states that:

Natural persons may insert their electronic health data into their own EHR or in that of natural persons whose health information they can access, through electronic health data access services or applications linked to these services. That information shall be marked as inserted by the natural person or by his or her representative [9].

### The Research

Although the Nordic countries are advanced in providing ORA, entering health data into the EHR is not widely implemented. Swedish patients could previously comment in their EHRs [63], but this function was removed in 2022 due to technical problems related to the initial implementation of the feature. In Finland, patients can save health data to their personal health record via well-being applications, but the function is in limited use, only certain applications are accepted, and the data are not yet available to health care professionals [64]. Furthermore, patients in both Finland and Sweden have asked for more interactivity in their health records, such as the possibility to comment on the notes or request corrections [4,6].

In the NORDeHEALTH project, we explore how patient input to the EHR might become better designed to adequately meet this function. The EHR has traditionally been available to health care professionals only. Patients report many positive effects from accessing their records, yet to fully achieve the potential benefits of digitalization, we need to further explore how EHRs can shift from being solely a documentation tool for health professionals to a tool for secure collaboration and communication with patients and family caregivers. Here, national patient portals and additional digital services will complement the future development of the EHRs into collaborative, person-centered tools.

### Viewpoint

As digitalization is about more than making electronic versions of analog work, we investigate different ways to use the power of digitalization, as provided in Textbox 3.

**Different ways to incorporate patient input to the electronic health record (EHR).**

- Patient input to the EHR in narrative form, for example, patients commenting on notes or contributing with descriptions of their symptoms.
- Patient-created structured data, for example, patient-reported outcome or experience measures—patient reported outcome measures and patient reported experience measures, and Integration of data and services from third-party applications, for example, self-tracking data, and decision support.

## Access Control

### The Proposal

Finally, EHDS proposes increased access control for patients. Patients should be able to request that electronic health data are made accessible to actors in the health or social security sector, and they should also have the right to restrict such access to electronic health data.

Natural persons shall have the right to give access to or request a data holder from the health or social security sector to transmit their electronic health data to a data recipient of their choice from the health or social security sector, immediately, free of charge, and without hindrance from the data holder or from the manufacturers of the system used by that holder [Article 3, §8] [9]

[...] natural persons shall have the right to restrict access of health professionals to all or part of their electronic health data [Article 3, §9] [9]

### The Research

The Swedish Agency for Health and Care Services Analysis [65] reports that the majority of the population accepts and wants digital data about their own care and health to be used so that it is useful, including for safer care and research. At the same time, it is important that the data be handled securely and protected from unauthorized access.

In the NORDeHEALTH 2022 patient survey [37], questions related to sharing of the respondents’ records were asked, as well as requests for unwanted record access. Among the Swedish respondents, 4% (501/12,334) of respondents answered that they had experienced that someone had seen their health records without their consent [66], a finding that requires further analysis. Although 4% can be considered a low number, it stands in stark contrast to the clear message from policy makers that unauthorized access should not occur [9,50].

With the increasing possibilities for secondary use of health data, both by research and industry stakeholders, it will become even more important for patients to be aware of how to manage their health data, and consent to sharing it, in a safe way. Further research is needed both to understand patients' incentives for and experiences of sharing their data for secondary use and to identify interventions to increase digital health literacy regarding secondary use specifically.

### Viewpoint

Unauthorized access can reduce both patient safety and trust in health care, but it can also erode opportunities for secondary use of health data. Existing controls for information security and privacy, therefore, need to be improved in line with the EHDS proposal.

## Conclusions

We argue that with the realization of the EHDS, patients’ opportunities to access and control third-party access to their EHRs are likely to change dramatically. ORA implementation today is fragmented throughout Europe, and the EHDS proposal aims to ensure all European citizens have equal online access to their health data. However, we argue that in order to implement the EHDS, we need more research evidence on the key ORA principles we have identified in our analysis. Results from the NORDeHEALTH project provide some of that evidence, but we have also identified important knowledge gaps that still need further exploration. Research such as that performed in the NORDeHEALTH project offers important firsthand insights and will be essential to inform the design and implementation of ORA to meet the requirements of the EHDS. However, further international collaboration and research, and dedicated funding, are needed to achieve a comprehensive understanding of sociotechnical and contextual factors necessary to consider for ensuring successful, secure, and ethical implementation of EHDS.

## Abbreviations

EHDS: European Health Data Space
EHR: electronic health record
NORDeHEALTH: Nordic eHealth for Patients: Benchmarking and Developing for the Future
ORA: online record access
PAEHR: patient accessible electronic health record

## Data Availability

Data sharing is not applicable to this article as no data sets were generated or analyzed during this study.

## References

[1] Holmgren AJ, Downing NL, Tang M, Sharp C, Longhurst C, Huckman RS (2022). Assessing the impact of the COVID-19 pandemic on clinician ambulatory electronic health record use. J Am Med Inform Assoc.

[2] Hägglund M, McMillan B, Whittaker R, Blease C (2022). Patient empowerment through online access to health records. Br Med J.

[3] Maria Hägglund: Nordic countries lead new initiative on patient access to EHRs. Br Med J.

[4] Moll J, Rexhepi H, Grünloh C, Huvila I, Hägglund M, Myreteg G, Scandurra I, Åhlfeldt RM, Cajander (2018). Patients' experiences of accessing their Electronic Health Records: National Patient Survey in Sweden. J Med Internet Res.

[5] Zanaboni P, Kummervold PE, Sørensen T, Johansen MA (2020). Patient use and experience with online access to Electronic Health Records in Norway: results from an online Survey. J Med Internet Res.

[6] Kujala S, Hörhammer Iiris, Väyrynen A, Holmroos M, Nättiaho-Rönnholm M, Hägglund M, Johansen MA (2022). Patients' experiences of web-based access to Electronic Health Records in Finland: cross-sectional Survey. J Med Internet Res.

[7] Salmi L, Blease C, Hägglund M, Walker J, DesRoches CM (2021). US policy requires immediate release of records to patients. Br Med J.

[8] Digital health platform handbook: building a digital information infrastructure (‎infostructure)‎ for health. World Health Organization, Union IT.

[9] European Health Union: a European Health Data Space for people and science. European Commission.

[10] Blease C, Torous J, Dong Z, Davidge G, DesRoches C, Kharko A, Turner A, Jones R, Hägglund M, McMillan B (2023). Patient online record access in English primary care: qualitative survey study of general practitioners' views. J Med Internet Res.

[11] Hägglund M, Scandurra I (2017). Patients' online access to electronic health records: current status and experiences from the implementation in Sweden. Stud Health Technol Inform.

[12] Walker J, Leveille S, Bell S, Chimowitz H, Dong Z, Elmore JG, Fernandez L, Fossa A, Gerard M, Fitzgerald P, Harcourt K, Jackson S, Payne TH, Perez J, Shucard H, Stametz R, DesRoches C, Delbanco T (2019). OpenNotes after 7 years: patient experiences with ongoing access to their clinicians' Outpatient Visit Notes. J Med Internet Res.

[13] Neves AL, Freise L, Laranjo L, Carter AW, Darzi A, Mayer E (2020). Impact of providing patients access to electronic health records on quality and safety of care: a systematic review and meta-analysis. BMJ Qual Saf.

[14] Kharko A, Blease C, Johansen M, Moen A, Scandurra I, McMillan B, Hägglund M (2024). Mapping patients' online record access worldwide: preliminary results from an international survey of healthcare experts. Stud Health Technol Inform.

[15] Directive (EU) 2022/2555 of the European Parliament and of the Council of 14 December 2022 on measures for a high common level of cybersecurity across the Union, amending Regulation (EU) No 910/2014 and Directive (EU) 2018/1972, and repealing Directive (EU) 2016/1148 (NIS 2 Directive).

[16] Essén A, Scandurra I, Gerrits R, Humphrey G, Johansen MA, Kierkegaard P, Koskinen J, Liaw S, Odeh S, Ross P, Ancker Js (2018). Patient access to electronic health records: Differences across ten countries. Health Policy and Technology.

[17] Cijvat CD, Cornet R, Hägglund M (2021). Factors influencing development and implementation of patients' access to electronic health records—a comparative study of Sweden and the Netherlands. Front Public Health.

[18] Kainiemi E, Vehko T, Kyytsönen M, Hörhammer I, Kujala S, Jormanainen V, Heponiemi T (2022). The factors associated with nonuse of and dissatisfaction with the National Patient Portal in Finland in the era of COVID-19: population-based cross-sectional survey. JMIR Med Inform.

[19] Petersson L, Erlingsdóttir G (2018). Open notes in Swedish Psychiatric Care (Part 2): survey among psychiatric care professionals. JMIR Ment Health.

[20] Grünloh C, Myreteg G, Cajander A, Rexhepi H (2018). "Why Do They Need to Check Me?" Patient participation through eHealth and the doctor-patient relationship: qualitative study. J Med Internet Res.

[21] Grünloh C, Cajander Å, Myreteg G (2016). "The Record is Our Work Tool!"-physicians' framing of a patient portal in Sweden. J Med Internet Res.

[22] Kristiansen E, Johansen M, Zanaboni P (2019). Healthcare personnels’ experience with patients’ online access to electronic health records: differences between professions, regions, and somatic and psychiatric healthcare. https://ep.liu.se/en/conference-article.aspx?series=&issue=161&Article_No=16.

[23] Johansen MA, Kummervold PE, Sørensen T, Zanaboni P (2019). Health professionals' experience with patients accessing their electronic health records: results from an online survey. Stud Health Technol Inform.

[24] Petersson L, Erlingsdóttir G (2018). Open notes in Swedish Psychiatric Care (Part 1): survey among psychiatric care professionals. JMIR Ment Health.

[25] Hagström J, Scandurra I, Moll J, Blease CR, Haage B, Hörhammer I, Hägglund M (2022). Minor and parental access to electronic health records: differences across four countries. Stud Health Technol Inform.

[26] Blease C, Torous J, Kharko A, DesRoches CM, Harcourt K, O'Neill S, Salmi L, Wachenheim D, Hägglund M (2021). Preparing patients and clinicians for Open Notes in Mental Health: qualitative inquiry of international experts. JMIR Ment Health.

[27] Blease CR, O'Neill SF, Torous J, DesRoches CM, Hägglund M (2021). Patient access to mental health notes: motivating evidence-informed ethical guidelines. J Nerv Ment Dis.

[28] Blease C, Salmi L, Rexhepi H, Hägglund M, DesRoches CM (2021). Patients, clinicians and open notes: information blocking as a case of epistemic injustice. J Med Ethics.

[29] Hägglund Maria, Scandurra I (2017). A socio-technical analysis of patient accessible electronic health records. Stud Health Technol Inform.

[30] Commission welcomes European Parliament's adoption of the European Health Data Space and regulation on substances of human origin. European Commission.

[31] Sittig DF, Singh H (2010). A new sociotechnical model for studying health information technology in complex adaptive healthcare systems. Qual Saf Health Care.

[32] Bärkås A, Scandurra I, Rexhepi H, Blease C, Cajander Å, Hägglund M (2021). Patients' access to their psychiatric notes: current policies and practices in Sweden. Int J Environ Res Public Health.

[33] Bärkås A, Hägglund M, Moll J, Cajander Å, Rexhepi H, Hörhammer I, Blease C, Scandurra I (2022). Patients' access to their Psychiatric Records—a comparison of four countries. Stud Health Technol Inform.

[34] Schwarz J, Bärkås A, Blease C, Collins L, Hägglund M, Markham S, Hochwarter S (2021). Sharing clinical notes and Electronic Health Records with people affected by mental health conditions: scoping review. JMIR Ment Health.

[35] Hagström J, Blease C, Haage B, Scandurra I, Hansson S, Hägglund M (2022). Views, use, and experiences of web-based access to pediatric electronic health records for children, adolescents, and parents: scoping review. J Med Internet Res.

[36] Simola S, Hörhammer I, Xu Y, Bärkås A, Fagerlund AJ, Hagström J, Holmroos M, Hägglund M, Johansen MA, Kane B, Kharko A, Scandurra I, Kujala S (2023). Patients' experiences of a National Patient Portal and its usability: cross-sectional survey study. J Med Internet Res.

[37] Hägglund M, Kharko A, Hagström J, Bärkås A, Blease C, Cajander Å, DesRoches C, Fagerlund A, Haage B, Huvila I, Hörhammer I, Kane B, Klein G, Kristiansen E, Luks K, Moll J, Muli I, Raphaug EH, Rexhepi H, Riggare S, Ross P, Scandurra I, Simola S, Soone H, Wang B, Ghorbanian Zolbin M, Åhlfeldt RM, Kujala S, Johansen MA (2023). The NORDeHEALTH 2022 Patient Survey: cross-sectional study of National Patient Portal users in Norway, Sweden, Finland, and Estonia. J Med Internet Res.

[38] (2001). HOD, The Norwegian Patient Right Act.

[39] (2012). Én inbygger - én journal? [in Norwegian] Meld. St. 9 (2012–2013).

[40] Perlich A, Meinel C (2015). Automatic treatment session summaries in psychotherapy—a step towards therapist-patient cooperation. Procedia Comput Sci.

[41] Kaihlanen AM, Virtanen L, Buchert U, Safarov N, Valkonen P, Hietapakka L, Hörhammer I, Kujala S, Kouvonen A, Heponiemi T (2022). Towards digital health equity—a qualitative study of the challenges experienced by vulnerable groups in using digital health services in the COVID-19 era. BMC Health Serv Res.

[42] Barnett K, Mercer SW, Norbury M, Watt G, Wyke S, Guthrie B (2012). Epidemiology of multimorbidity and implications for health care, research, and medical education: a cross-sectional study. Lancet.

[43] Chou AF, Stewart SL, Wild RC, Bloom JR (2012). Social support and survival in young women with breast carcinoma. Psychooncology.

[44] Goren A, Gilloteau I, Lees M, DaCosta Dibonaventura M (2014). Quantifying the burden of informal caregiving for patients with cancer in Europe. Support Care Cancer.

[45] Braun M, Mikulincer M, Rydall A, Walsh A, Rodin G (2007). Hidden morbidity in cancer: spouse caregivers. J Clin Oncol.

[46] Adelman RD, Tmanova LL, Delgado D, Dion S, Lachs MS (2014). Caregiver burden: a clinical review. J Am Med Assoc.

[47] Wolff JL, Darer JD, Berger A, Clarke D, Green JA, Stametz RA, Delbanco T, Walker J (2016). Inviting patients and care partners to read doctors' notes: OpenNotes and shared access to electronic medical records. J Am Med Inform Assoc.

[48] Latulipe C, Mazumder SF, Wilson RKW, Talton JW, Bertoni AG, Quandt SA, Arcury TA, Miller DP (2020). Security and privacy risks associated with adult patient portal accounts in US hospitals. JAMA Intern Med.

[49] Nurgalieva L, Cajander Å, Moll J, Åhlfeldt RM, Huvila I, Marchese M (2020). 'I do not share it with others. No, it's for me, it's my care': on sharing of patient accessible electronic health records. Health Informatics J.

[50] Lag (2018:218) med kompletterande bestämmelser till EU:s dataskyddsförordning.

[51] Bell SK, Folcarelli P, Fossa A, Gerard M, Harper M, Leveille S, Moore C, Sands KE, Sarnoff Lee B, Walker J, Bourgeois F (2021). Tackling ambulatory safety risks through patient engagement: what 10,000 patients and families say about safety-related knowledge, behaviors, and attitudes after reading visit notes. J Patient Saf.

[52] Newman-Toker DE, Pronovost PJ (2009). Diagnostic errors—the next frontier for patient safety. J Am Med Assoc.

[53] Singh H, Meyer AND, Thomas EJ (2014). The frequency of diagnostic errors in outpatient care: estimations from three large observational studies involving US adult populations. BMJ Qual Saf.

[54] Singh H, Giardina TD, Meyer AND, Forjuoh SN, Reis MD, Thomas EJ (2013). Types and origins of diagnostic errors in primary care settings. JAMA Intern Med.

[55] Singh H, Hirani K, Kadiyala H, Rudomiotov O, Davis T, Khan MM, Wahls TL (2010). Characteristics and predictors of missed opportunities in lung cancer diagnosis: an electronic health record-based study. J Clin Oncol.

[56] Singh H, Daci K, Petersen LA, Collins C, Petersen NJ, Shethia A, El-Serag HB (2009). Missed opportunities to initiate endoscopic evaluation for colorectal cancer diagnosis. Am J Gastroenterol.

[57] Balogh EP, Miller BT, Ball JR (2015). Improving Diagnosis in Health Care.

[58] Gandhi TK, Kaplan GS, Leape L, Berwick DM, Edgman-Levitan S, Edmondson A, Meyer GS, Michaels D, Morath JM, Vincent C, Wachter R (2018). Transforming concepts in patient safety: a progress report. BMJ Qual Saf.

[59] Blease CR, Bell SK (2019). Patients as diagnostic collaborators: sharing visit notes to promote accuracy and safety. Diagnosis (Berl).

[60] Tapuria A, Porat T, Kalra D, Dsouza G, Xiaohui S, Curcin V (2021). Impact of patient access to their electronic health record: systematic review. Inform Health Soc Care.

[61] Blease C, Cohen IG, Hoffman S (2022). Sharing clinical notes: potential medical-legal benefits and risks. J Am Med Assoc.

[62] Bärkås A, Kharko A, Blease C, Cajander Å, Johansen Fagerlund A, Huvila I, Johansen MA, Kane B, Kujala S, Moll J, Rexhepi H, Scandurra I, Wang B, Hägglund M (2023). Errors, omissions, and offenses in the health record of mental health care patients: results from a nationwide survey in Sweden. J Med Internet Res.

[63] Hägglund M (2017). Electronic health records in Sweden—how can we go from transparency to collaboration?. BMJ Opinion.

[64] Jormanainen V (2018). Large-scale implementation and adoption of the Finnish national Kanta services in 2010–2017: a prospective, longitudinal, indicator-based study. FinJeHeW.

[65] (2017). For Safety's Sake: The population's attitude to the benefits and risks of digital health data [in Swedish]. Swedish Agency for Health and Care Services Analysis.

[66] Bärkås A, Kharko A, Åhlfeldt RM, Hägglund M (2023). Patients' experiences of unwanted access to their online health records. Stud Health Technol Inform.

